# Prediction of wheat gluten composition via near-infrared spectroscopy

**DOI:** 10.1016/j.crfs.2023.100471

**Published:** 2023-03-02

**Authors:** Clemens Schuster, Julien Huen, Katharina Anne Scherf

**Affiliations:** a Leibniz-Institute for Food Systems Biology at the Technical University of Munich, Lise-Meitner-Str. 34, 85354, Freising, Germany; b Technologie-Transfer-Zentrum ttz Bremerhaven, Am Lunedeich 12, 27572, Bremerhaven, Germany; c Department of Bioactive and Functional Food Chemistry, Institute of Applied Biosciences, Karlsruhe Institute of Technology (KIT), Adenauerring 20a, 76131, Karlsruhe, Germany

**Keywords:** Baking quality, Gliadin, Glutenin, NIRS, Reversed-phase high-performance liquid chromatography (RP-HPLC), PLS-regression

## Abstract

Gluten composition is an important quality parameter for wheat flour, because it is strongly correlated to baking quality. Wheat proteins are commonly extracted stepwise and analysed using RP-HPLC-UV to determine the gluten composition. This procedure is very time-consuming and labour-intensive. Therefore, a new, fast and easy method to quantitate gluten proteins was established using NIR spectroscopy (NIRS). PLS-regression models were calculated containing 207 samples for calibration and 169 for test set validation. Albumin/globulin (ALGL), gluten, gliadin and glutenin content was predicted with a root mean square error of prediction (RMSEP) of 2.01 mg/g, 6.09 mg/g, 4.25 mg/g and 3.50 mg/g, respectively. High-molecular-weight glutenin subunits (HMW-GS) and low-molecular-weight glutenin subunits (LMW-GS) were predicted with a RMSEP of 1.12 mg/g and 2.38 mg/g. The relative error was too high for ALGL, LMW-GS and HMW-GS, but that of gluten, gliadins and glutenins was in a range comparable to the reference method. Therefore, the new NIRS method can be used to estimate the gluten composition of wheat flour, including the gliadin/glutenin and the LMW-GS/HMW-GS ratio.

## Highlights

• Quantitation of gluten, gliadin and glutenin for quality assessment of wheat flour.
• NIR spectroscopy is a fast and easy approach to quantitate gluten proteins.
• PLS regression to predict gluten, gliadin and glutenin content.
• NIRS is a suitable fast method to determine the gluten composition of wheat flour.

## Graphical abstract

**Figure:**
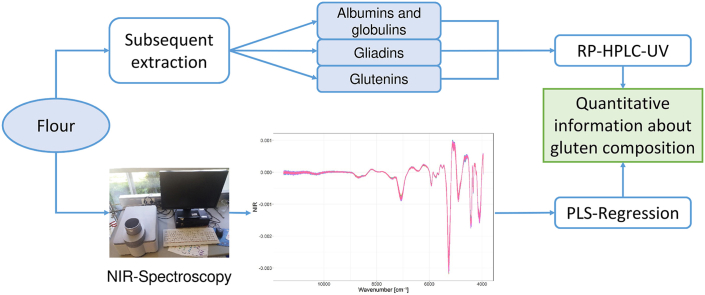

## 1 Introduction

The baking quality of wheat flour is widely associated with protein content, especially gluten proteins (Don et al., 2003; Goesaert et al., 2005; Payne et al., 1987). The Dumas combustion method (ICC standard No. 167, 2000) or Kjeldahl method (ICC standard No. 105/2, 1994) are well established for the quantitation of crude protein in wheat flour. However, protein quantity is often not sufficient as a predictor for baking quality, because different baking quality was observed for flours with comparable protein content, but different gluten composition (Gabriel et al., 2017). Payne et al. (1987) already found that different alleles for high-molecular-weight glutenin subunits (HMW-GS) were associated with baking quality. Recent studies also showed that changes in gluten composition had a larger influence on baking quality than the increase of protein content (Rossmann et al., 2020; Xue et al., 2016). Therefore, knowledge about the composition of gluten is important throughout the value chain of wheat flour.

Identification and quantitation of gluten proteins is often performed using a combination of extraction and chromatographic separation. Following a modified version of the Osborne fractionation, albumins and globulins (ALGL), gliadins and glutenins are extracted from the flour in three steps and analysed by RP -HPLC with UV-detection (Wieser et al., 1998). The protein content is then calculated as the sum of all three protein fractions quantitated with RP-HPLC. Advantages of this method are that it requires only small amounts of flour (100 mg), shows good precision (relative standard deviation of less than 10%, n = 3) and provides detailed information about gluten composition (Pronin et al., 2020; Schuster et al., 2023). However, it also has limitations, because it is destructive, very time-consuming, labour-intensive and comparatively expensive considering the costs of solvents, columns and the HPLC instrument.

As gluten composition is an important quality trait, fast and easy methods for its determination are desirable. Near-infrared spectroscopy (NIRS) is a well-established, quick and comparatively cheap technique in food analytics (Cen and He, 2007; Osborne et al., 1982). To quantitate a parameter via NIRS requires a multivariate calibration model to correlate spectral structures to the analytical values as a target value (Geladi, 2003). Regarding flour characteristics, partial least squares (PLS)-regression models were successfully calibrated for several indirect quality traits like crude protein (ICC Standard No. 159, 1995), dry and wet gluten content, Farinograph and Alveograph parameters and Zeleny sedimentation value (Barak et al., 2013; Chen et al., 2017; Miralbés, 2003). Most research focuses on the prediction of rheological quality parameters which were related to baking quality, or on the crude protein and wet and dry gluten content. In addition, NIRS was already used as a tool to identify flour batches which are not suitable for bread making (Li Vigni et al., 2009). First experiments were already made to predict gliadin and glutenin content using NIRS (Delwiche et al., 1998; Wesley et al., 2001). Both studies used size exclusion (SE)-HPLC as a reference method to determine gliadin and glutenin content. SE-HPLC separates proteins according to their molecular weight, which is continuous and overlaps to a certain degree for gliadin and glutenin protein types. Furthermore, gluten protein types (i.e., α-, γ-, ω5-, ω1,2-, and ωb-gliadins, low-molecular-weight glutenin subunits (LMW-GS) and HMW-GS) were not considered in their approaches.

Based on the most recent findings on bread making properties and protein composition, gluten quality should be integrated into the quality assessment of wheat flour (Gabriel et al., 2017). Therefore, fast, easy and cost-effective methods are necessary to determine gluten quality. As only small sample amounts are available in the early stages of seed breeding, non-destructive methods such as NIRS are preferable. In this study, our aim was to calibrate PLS-regression models to predict gluten, gliadin, glutenin, HMW-GS and LMW-GS content using NIRS. Therefore, an extensive set of 376 wheat flour samples was analysed by NIRS and with RP-HPLC-UV as a reference method.

## 2 Materials and methods

### 2.1 Wheat samples

In total, 376 different wheat samples were included in this study. The sample set contained the 82 flour samples described in detail by Schuster et al. (2023) and 294 wheat samples provided by the Bavarian State Research Center for Agriculture, Institute for Crop Science and Plant Breeding (Freising, Germany) (Geyer et al., 2022; Stadlmeier et al., 2018). All samples were white flours of type 550 (according to the German flour classification system, i.e., ash content 0.51%–0.63% based on dry matter) with no additives. The flour samples were stored in closed polypropylene bottles under ambient conditions.

### *2.2* Crude protein content

Crude protein content was determined according to ICC Standard no 167 (2000). For the Dumas combustion method 50 mg of flour were weighed into a tin foil cup and analysed with a Leco TruSpec nitrogen analyser (Leco, Kirchheim, Germany). A conversion factor of 5.7 was used to calculate crude protein content.

### 2.3 Determination of wheat protein composition

Extraction and quantitation of wheat proteins was performed according to Wieser et al. (1998) with some modifications as described in Schuster et al. (2023). Three independent extracts of ALGL, gliadins and glutenins were analysed in RP-HPLC experiments for each flour sample. Calibration was performed by correlating the peak area to the protein content of the reference material of the Prolamin Working Group (van Eckert et al., 2006). The sum of gliadins and glutenins represents the gluten content.

### 2.4 NIR-spectroscopy

NIR spectra of the flours were measured using a Tango-R FT-NIR-spectrometer (Bruker Optics GmbH, Ettlingen, Germany) in reflectance mode. The Tango-R measures wavenumbers between 11,545 cm^−1^ and 3946 cm^−1^ (866 nm–2534 nm) with a resolution of 8 cm^−1^. The measurement spot of the spectrometer had a diameter of 10 mm. A rotating sample cup (diameter 97 mm) was used to maximize the analysed sample surface. The sample cup was filled with flour up to a height of approximately 4 cm. For each sample, 64 scans were measured. Background spectra were subtracted from sample spectra and measured every 10 samples.

Reference spectra of water, wheat starch (Kröner-Stärke GmbH, Ibbenbüren, Germany), vital gluten (Kröner-Stärke GmbH) and dried flour were measured to assign NIR absorption bands to flour constituents.

### 2.5 Data analysis

NIR spectra were measured using the OPUS 7.8 software (Bruker Optics GmbH, Ettlingen, Germany). Raw files were exported and further analysed in R using the pls package for PLS-regression (Mevik and Wehrens, 2007). As described by Rinnan et al. (2009), the most common methods for data pre-processing of NIR spectra were applied. Standard Normal Variate (SNV) was used for scatter correction, which also leads to an adjustment of baseline shifts. For calculations of first and second derivative of spectra, the Savitzky-Golay filter (polynomial order: 2; smoothing points: 17) was used. The combination of SNV and first derivative was tested as well. Data pre-treatment was performed in OPUS 7.8. For the development of regression models, leave-one-out cross validation was used. The number of factors was chosen by minimizing the root mean square error of cross validation (RMSECV) and maximizing the explained variance. A permutation test was performed to prevent overfitting. The dataset was randomly divided into calibration data (207 samples) and test data (169 samples). Samples with minimal and maximal gluten content were assigned to the calibration data to maximize the calibrated range of PLS-regression models.

## 3 Results & discussion

### 3.1 Wheat flour protein composition

The gluten, gliadin and glutenin content of the sample set containing 376 white wheat flours ranged from 47.7 mg/g to 132.7 mg/g, 32.1 mg/g to 95.3 mg/g and 15.6 mg/g to 46.2 mg/g, respectively (Table 1). The ratio of gliadins to glutenins was between 1.3 and 2.9, which is a typical range for wheat (Marti et al., 2015). The moisture content of the analysed wheat flours ranged from 9.6 to 14.0 g/100g (median 12.8 g/100g).

**Table 1:** Content of wheat protein fractions determined by RP-HPLC in all 376 samples.

Parameter	Range [mg/g]	Median [mg/g]
*ALGL*	5.6–20.2	14.1
*Gluten*	47.7–132.7	77.1
*Gliadins*	32.1–95.3	50.5
*Glutenins*	15.6–46.2	26.5
*Gliadin/glutenin ratio*	1.3–2.9	1.9
*LMW-GS/HMW-GS ratio*	1.8–3.5	2.5

ALGL, Albumins/globulins; LMW-GS/HMW-GS, ratio of low-molecular-weight glutenin subunits to high molecular weight glutenin subunits; all values based on flour weight.

**Fig. 1:**
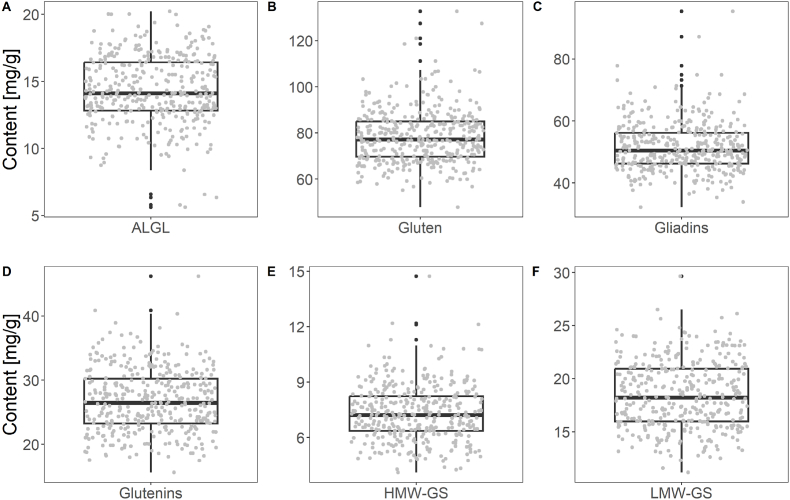
Box and whisker plots for the content of albumins and globulins (ALGL) (A), gluten (B), gliadins (C), glutenins (D), high-molecular-weight glutenin subunits (HMW-GS) (E) and low-molecular-weight glutenin subunits (LMW-GS) (F). Boxes represent the interquartile range, the line in the box indicates the median and whiskers represent the lower quartile minus 1.5 times the interquartile range and the upper quartile plus 1.5 times the interquartile range, respectively. Values below or above the whiskers (black dots) were therefore marked as outliers.

To generate reliable calibration models, the sample set needs to consist of representative samples. Box and whisker plots of the respective analytes show an even distribution of samples within the interquartile range (Fig. 1). Samples are marked as possible outliers if they exceeded the distance of 1.5 times the interquartile range above the upper quartile or below the lower quartile. Within the sample set, four samples had lower a ALGL content than the described range. For gluten, gliadins, glutenins, HMW-GS and LMW-GS, there was at least one sample with a content above this range (Fig. 1). Therefore, the prediction of the regression models was less precise within this range, because only few samples determined the slope of the regression line in this area. These samples were included anyway to maximize the calibrated range. For further method optimization, additional samples with low ALGL content and high gluten, gliadin, glutenin, HMW-GS and LMW-GS contents should be included to the calibration data.

### 3.2 Spectral characterization

**Fig. 2:**
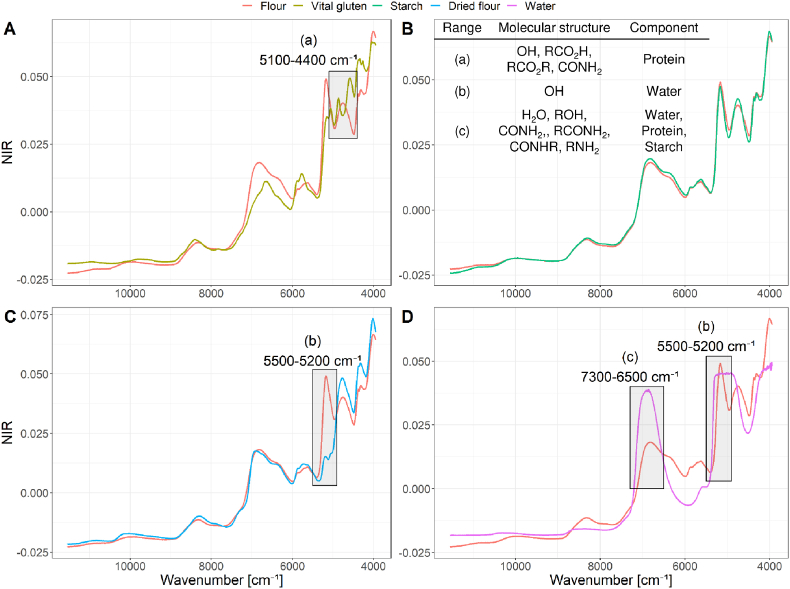
Normalized (standard normal variate) NIR spectra of a native flour sample compared to vital gluten (A), starch (B), a dried flour sample (C) and water (D), respectively. The dried flour sample is the same as the native flour. Boxes indicate relevant wavenumbers that show the most pronounced differences.

**Fig. 3:**
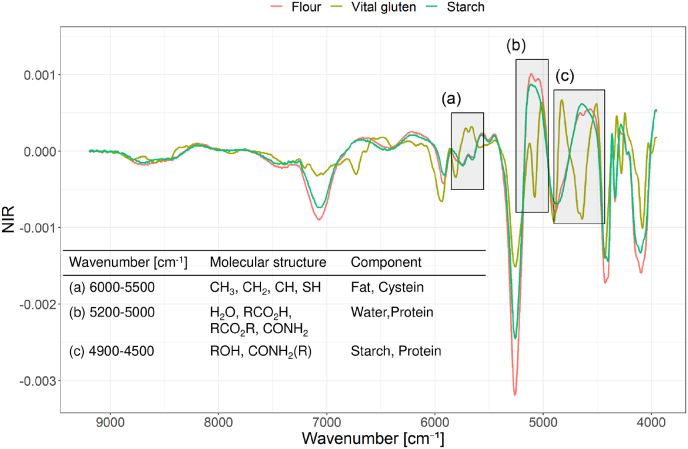
First derivative (Savitzky-Golay filter; polynomial order: 2, smoothing points: 9) of the NIR spectra of a flour sample, vital gluten and wheat starch. Boxes indicate relevant wavenumbers.

NIR spectra of one representative wheat flour sample (commercial flour sample of type 550 received from Dresdener Mühle GmbH (Dresden, Germany)) were analysed and compared to vital gluten (protein content 71.3 g/100g), wheat starch, water and the same flour sample dried to a moisture content of 1% to identify relevant wavenumbers for protein absorptions (Fig. 2). Absorption bands around 6840 cm^−1^ and 5160 cm^−1^ were assigned to O–H bonds of water (Li Vigni et al., 2009). After drying the flour sample, the absorption band at 5160 cm^−1^ disappeared and a clear assignment was possible. Spectra of wheat starch and native flour showed only small differences (Fig. 2 B). Flour contains approximately 70–75% of starch (Goesaert et al., 2005) and therefore great similarities within the NIR spectra of wheat starch and flour were expected. Protein absorptions should be exposed in the spectra of a vital gluten sample containing approximately 70% of gluten proteins. NIR spectra of flour and vital gluten also showed comparable absorption bands. Differences were observed between 6000 cm^−1^ to 5500 cm^−1^, as well as between 5100 cm^−1^ to 4900 cm^−1^ and at 4400 cm^−1^. The spectrum of vital gluten showed two maxima at 4860 cm^−1^ and 4580 cm^−1^ whereas the flour spectrum showed only one maximum at 4750 cm^−1^. Savitzky-Golay derivation was used to focus on flat structures within the spectra. The first derivative of the spectra of vital gluten showed maxima at 4825 cm^−1^ and 4520 cm^−1^, which were also present as shoulders of the absorption band at 4600 cm^−1^ in the flour spectrum. The spectrum of wheat starch did not show these shoulders (Fig. 3). Peaks at 5130 cm^−1^ and 5017 cm^−1^ were present in the spectra of vital gluten and flour, but not in the spectrum of wheat starch. The extracted wavenumbers relevant for protein absorption were in accordance to literature (Cen and He, 2007; Salgó and Gergely, 2012). Spectral ranges containing most information about proteins were taken into account for optimization of the following PLS-regressions.

### 3.3 PLS-regression

**Fig. 4:**
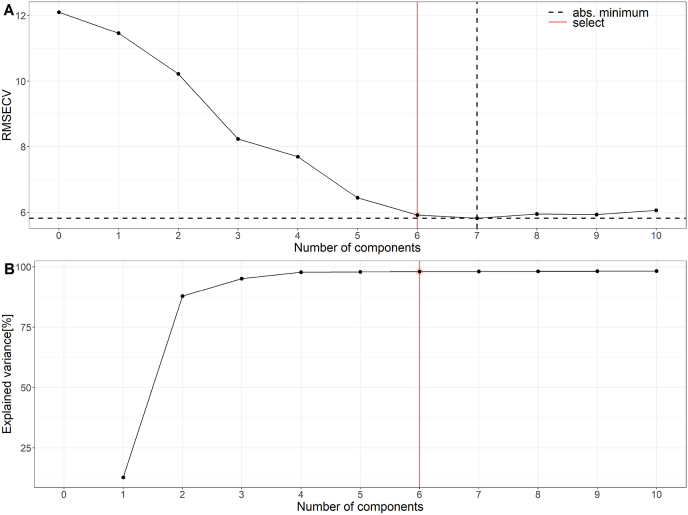
RMSECV (A) and explained variance in percent (B) for the respective number of components for the PLS-regression model to predict the gluten content.

**Fig. 5:**
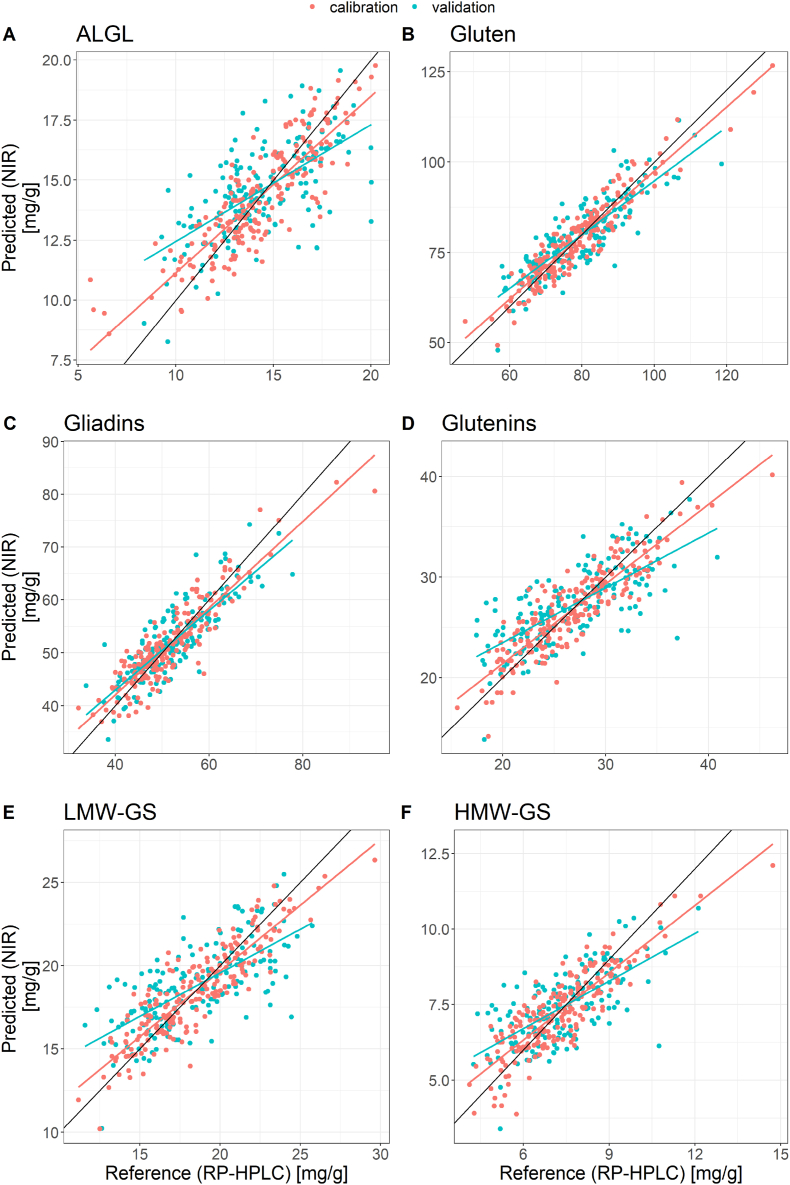
Comparison of predicted and reference values for the content of albumins and globulins (ALGL) (A), gluten (B), gliadins (C), glutenins (D), low-molecular-weight glutenin subunits (LMW-GS) (E) and high-molecular-weight glutenin subunits (HMW-GS) (F) of the calibration (n = 207) and validation (n = 169) data set.

PLS-regression models were calculated for the quantitation of gluten proteins. For method development, different spectral ranges and pre-processing options were tested and PLS-regression models were calculated with different combinations of pre-processed spectra and spectral ranges. Leave-one-out cross validation was used to estimate the quality of the regression. Optimization of the regression models was performed by minimizing the RMSECV. To avoid overfitting, the number of PLS factors was chosen according to a permutation test. The test aims to choose a minimal RMSECV with maximal explained variance (shown for gluten as an example in Fig. 4). The model performance was determined by prediction of the target values of an independent test set (169 samples). A minimal root mean square error of prediction (RMSEP) was observed for the optimized PLS-regression models. Robust models were obtained which were characterized by comparable regression lines of the calibration data and the test data (Fig. 5). The best results to predict ALGL, gluten, gliadins, glutenins, HMW-GS and LMW-GS were obtained using the first derivative of the NIR spectra (Table 2). Even though wavenumbers of protein absorptions were identified within the NIR spectra, the best PLS-regression models were obtained using the complete spectral range from 11,545 cm^−1^ to 3946 cm^−1^.

**Table 2:** Best results for method development to calculate PLS-regression models to predict gluten composition.

Analyte	Spectral range [cm^−1^]	Data pretreatment	RMSECV [mg/g]	RMSEP [mg/g]	Number of factors
ALGL	11,545–3946	1. derivative	2.06	2.01	6
Gluten	11,545–3946	1. derivative	5.92	6.09	6
Gliadins	11,545–3946	1. derivative	4.43	4.25	5
Glutenins	11,545–3946	1. derivative	3.32	3.50	6
HMW-GS	11,545–3946	1. derivative	1.17	1.12	6
LMW-GS	11,545–3946	1. derivative	2.14	2.38	6
Gliadin/glutenin ratio	6181–3946	SNV +1. derivative	0.28	0.27	6
LMW-GS/HMW-GS ratio	6181–3946	SNV +1. derivative	0.28	0.27	6

RMSECV, root mean square error of cross validation; RMSEP, root mean square error of prediction; ALGL, albumins/globulins; HMW-GS, high-molecular-weight glutenin subunits; LMW-GS, low-molecular-weight glutenin subunits; SNV, standard normal variate; LMW-GS/HMW-GS, ratio of low-molecular-weight glutenin subunits to high molecular weight glutenin subunits.

For RP-HPLC as the reference method, a relative standard deviation of lower than 10% is tolerable for three independent replicates (Schuster et al., 2023; Schopf et al., 2021). Therefore, PLS-regression models with an RMSEP lower than 10% were considered to be acceptable. The PLS-regression for predicting the gluten content had an RMSEP of 6.09 mg/g, which implies a relative error of 10.7% for minimal and 5.1% for maximal gluten contents. Regressions for gliadins and glutenins had an RMSEP of 4.25 mg/g and 3.50 mg/g, respectively. These absolute errors correspond to a relative deviation of 5.5%–12.6% for gliadins and of 8.6%–20.1% for glutenins. Even if the errors are above 10% for small contents the models were considered acceptable. For ALGL (RMSEP = 2.01 mg/g), LMW-GS (RMSEP = 2.38 mg/g) and HMW-GS (RMSEP = 1.12 mg/g) the RMSEP led to a relative deviation of 10.0–24.0%, 9.2–20.6% and 9.2–26.4%, respectively. For ALGL, LMW-GS and HMW-GS, the errors regarding small contents were unacceptably high.

As described in 3.1, only a small number of samples within the sample set had high gluten (>119 mg/g), gliadin (>80 mg/g) or glutenin (>38 mg/g) contents. When comparing the predicted and the reference values for gluten and gliadins, the contents were underestimated in case of high amounts (Fig. 5 B and C). Samples with high glutenin content were both over- or underestimated and had an increased distance to the regression line. The same as for glutenins was observed for HMW-GS and LMW-GS (Fig. 5 E and F). Within the sample set, only a few samples showed contents lower than 8 mg/g of ALGL. Samples with low ALGL content tended to be overestimated (Fig. 5 A).

**Fig. 6:**
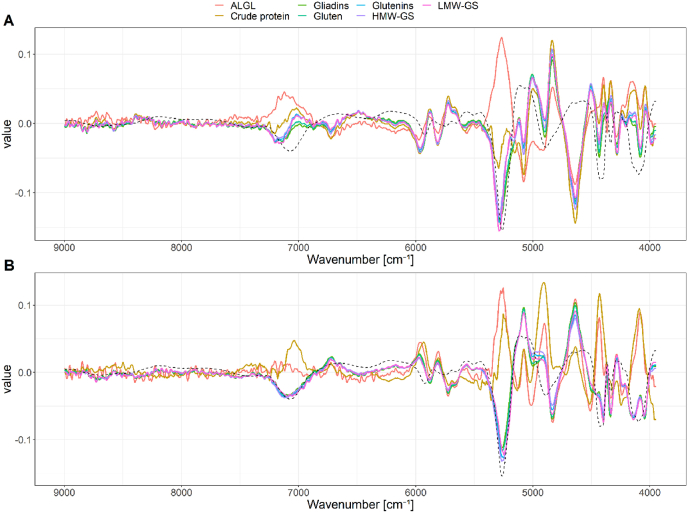
Loadings of PLS components 1 (A) and 2 (B) of PLS-regression models. The black dashed line is a flour spectrum (first derivative) as reference.

Prediction of the crude protein content using NIRS is already well-established (ICC standard No. 159, 1995; Osborne et al., 1982) and was also successfully done in this study (data not shown). Comparisons of the loadings of the regression models revealed that the same wavenumbers were relevant for each calibration (Fig. 6). It is likely that all regression models determined the crude protein content scaled to each component, as gluten (r = 0.87), gliadins (r = 0.82) and glutenins (r = 0.74) were highly correlated to the crude protein content. Wesley et al. (2001) showed that the prediction of gliadin and glutenin content using PLS-regressions is correlated to the total protein content. In their work only a classification of high, medium or low content of gliadins and glutenins was possible, as errors of regressions were unacceptably large for analytical purposes.

For ω5-, ω1,2-, α-, γ-, and ωb-gliadins the PLS-regression models had relative errors of up to 100% (data not shown) and were therefore not applicable for quantitation.

**Fig. 7:**
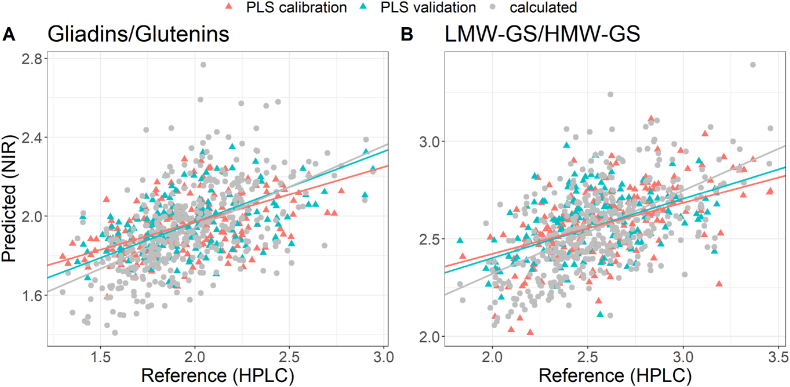
Comparison of predicted and reference values for gliadin/glutenin ratio (A) and LMW-GS/HMW-GS ratio (B) predicted by a PLS-regression model and calculated from the predicted content of gliadins, glutenins, LMW-GS and HMW-GS. Regression lines for calibration (red) and validation (blue) and calculated values (gray) are shown for PLS values. (For interpretation of the references to colour in this figure legend, the reader is referred to the Web version of this article.)

The ratios of gliadin/glutenin and LMW-GS/HMW-GS are known as important quality indicators for wheat flour (Barak et al., 2013; Gupta et al., 1992). Therefore, PLS-regressions were also calculated for these two parameters and a combination of SNV and first derivative showed the best results. In contrast to the other regression models, gliadin/glutenin and LMW-GS/HMW-GS ratio showed better results with selected spectral ranges. The PLS-regression model for predicting the gliadin/glutenin ratio included wavenumbers from 6181 to 3946 cm^−1^ whereas the regression model for LMW-GS/HMW-GS ratio included wavenumbers from 6181 to 5542 cm^−1^ and 5175 to 4436 cm^−1^, respectively. The RMSEP of the PLS-regression for predicting the gliadin/glutenin ratio was 0.27, which corresponds to a relative error of 9.2–19.6%. For the ratio of LMW-GS/HMW-GS, a RMSEP of 0.27 was observed (8.5–14.8%). It is questionable, whether distinct PLS-regressions for the gliadins/glutenins and LMW-GS/HMW-GS ratios are necessary, because gliadins, glutenins and LMW-GS were predicted with acceptable error. Therefore, we compared the ratios predicted by a distinct PLS-regression model with those calculated from the respective predicted contents (Fig. 7). Comparing the regression lines, almost no difference occurred and therefore an extra calibration to predict gliadin/glutenin ratio is not necessary. For the LMW-GS/HMW-GS ratio, a larger difference for regression lines was observed, which is due to the larger RMSEP of the regression model for HMW-GS.

## 4 Conclusion

Gluten quantity and quality are important parameters for the quality assessment of wheat flour. Using NIRS as a simple, fast (approximately 1 min per analysis), cost-effective and non-destructive analytical technique is desirable for predicting quality parameters of wheat flour. PLS-regression models with acceptable errors were calculated to predict gluten, gliadin and glutenin content and thus a fast and simple quantitation of important quality traits of wheat flour was possible for the first time. The error of prediction for ALGL, LMW-GS and HMW-GS was larger than the acceptable error of the reference method. Nevertheless, all of the presented PLS-regression models can be used if an estimation of the respective content is required. The gliadin/glutenin and LMW-GS/HMW-GS ratios were calculated with the predicted gliadin, glutenin, LMW-GS and HMW-GS contents with adequate accuracy. Therefore, NIRS is an appropriate fast method to determine the gluten composition of wheat flour.

## Funding

This IGF Project of the 10.13039/501100008465FEI was supported via AiF within the programme for promoting the Industrial Collective Research (IGF) of the Federal Ministry of Economic Affairs and Climate Action (BMWK), based on a resolution of the German Parliament. Project 20283 N. This work was additionally supported by funds of the 10.13039/501100005908Federal Ministry of Food and Agriculture (10.13039/501100005908BMEL) based on a decision of the Parliament of the Federal Republic of Germany via the Federal Office for Agriculture and Food (10.13039/501100010771BLE) under the innovation support programme, project BigBaking (2818404B18). We acknowledge support by the KIT-Publication Fund of the 10.13039/100009133Karlsruhe Institute of Technology.

## CRediT authorship contribution statement

**Clemens Schuster:** Conceptualization, Data curation, Formal analysis, Investigation, Methodology, Visualization, Writing – original draft. **Julien Huen:** Conceptualization, Funding acquisition, Resources, Writing – review & editing. **Katharina Anne Scherf:** Conceptualization, Funding acquisition, Resources, Supervision, Writing – review & editing.

## Declaration of competing interest

The authors declare that they have no known competing financial interests or personal relationships that could have appeared to influence the work reported in this paper.

## Data Availability

Data will be made available on request.
